# Fat grafting in soleal sling syndrome: a retrospective case series

**DOI:** 10.1080/23320885.2026.2715849

**Published:** 2026-08-19

**Authors:** Laura Hilbig-Vlatten, Miguel I. Dorante, Julius Asschenfeldt, Lilly F. Stadelmeier, Mario Keko, Arriyan S. Dowlatshahi

**Affiliations:** a Medical Faculty, University of Freiburg, Freiburg, Germany; b Division of Orthoplastic and Reconstructive Microsurgery, Beth Israel Deaconess Medical Center, Harvard Medical School, Boston, MA, USA; c Department of Orthopaedic Surgery, Beth Israel Deaconess Medical Center, Harvard Medical School, Boston, MA, USA; d Division of Plastic and Reconstructive Surgery, Lahey Hospital and Medical Center, UMass Chan Medical School, Burlington, MA, USA

**Keywords:** Fat grafting, soleal sling syndrome, peripheral nerve, lower extremity, tibial nerve, autologous fat grafting

## Abstract

Soleal sling syndrome (SSS) is an underrecognized cause of tibial nerve (TN) entrapment at the popliteal fossa, resulting in exertional calf pain, paresthesia and functional impairment. Five patients (seven limbs) underwent decompression with autologous fat grafting. Clinical improvement occurred without graft-related complications, supporting evaluation of fat grafting as an adjunctive treatment.

## Introduction

Soleal sling syndrome (SSS) is an underdiagnosed cause of tibial nerve (TN) entrapment at the popliteal fossa. It is characterized by TN compression beneath the tendinous arch of the soleus muscle, resulting in calf pain, numbness, tingling and weakness in the distal lower extremity [1]. Left untreated, SSS may lead to progressive neurologic impairment and functional limitation. Surgical decompression of the soleal sling remains the primary treatment and has been shown to result in symptom relief and improved quality of life (QoL) in most patients [2,3].

Despite technically adequate decompression, postoperative outcomes may be suboptimal in some cases. Perineural fibrosis, re-adhesion of the nerve to surrounding structures, and residual mechanical irritation may contribute to persistent pain, incomplete sensory or motor recovery, and recurrence of symptoms [4]. Perineural scarring remains a recognized challenge in peripheral nerve surgery and has prompted investigation into adjunctive strategies to improve the perineural environment.

In recent years, autologous fat grafting (AFG) has emerged as a potential adjunct in peripheral nerve surgery. Vaienti et al. demonstrated that fat grafts may function as a biological spacer, providing mechanical protection to the nerve and reducing tension and friction against adjacent tissues [5]. In addition, Podsednik et al. reported that adipose tissue contains mesenchymal stem cells with potential regenerative and angiogenic properties that may support nerve healing [6]. Krześniak and Noszczyk described favorable outcomes following fat grafting in recurrent compressive neuropathies, including revision carpal tunnel surgery [7].

Despite these applications, the role of AFG following soleal sling decompression has not been systematically explored and no established indication for its use in SSS currently exists. To our knowledge, no prior study has reported the technical feasibility and short-term safety of AFG in this setting. The objective of this case series is to describe our early experience with AFG applied at the time of soleal sling release in patients with SSS and to report postoperative symptom course and functional recovery.

## Patients and methods

### Study design and patient selection

This retrospective case series was conducted at a single academic center and included all patients who underwent soleal sling release with intraoperative AFG between January 2019 and September 2023. All procedures were performed by the senior author (ASD). Because no accepted diagnostic standard exists for SSS, patients were included based on a working diagnosis supported by exertional calf pain with plantar sensory symptoms, focal tenderness or Tinel’s sign at the soleal sling, magnetic resonance (MR) neurography and intraoperative evidence of TN compression. Electrodiagnostic studies (electromyography and electroneurography) were performed in all patients and were negative. Response to targeted botulinum toxin injection or ultrasound-guided hydrodissection was considered supportive when performed. Dynamic duplex ultrasound with resisted plantarflexion was used to assess concomitant popliteal artery entrapment syndrome (PAES); when positive, symptoms were considered potentially mixed neurovascular in origin and both PAES and SSS were addressed during the same operation. During the study period, adjunctive AFG was used as a routine component of soleal sling decompression by the senior author. There were no exclusions within the study period.

The study was approved by the Institutional Review Board (approval no. 2025P000359) and conducted in accordance with the Declaration of Helsinki. Written informed consent to publish clinical details and images was obtained from all subjects.

### Data collection

Demographic data (age, sex, race, body mass index), clinical characteristics (symptom duration, prior treatments, laterality), operative details (fat graft donor site and graft volume) and postoperative follow-up information were extracted from the electronic medical record. Data were stored and managed using REDCap, a secure, web-based research data platform [8].

Patient-reported outcome measures (PROMIS Physical Function and Pain Interference) were collected when available as part of routine clinical care. PROMIS scores were collected as patient-level measures and were not obtained at standardized postoperative intervals. Demographic variables are reported at the patient level (*n* = 5), while operative and clinical characteristics are reported at the limb/procedure level (*n* = 7).

### Surgical technique

All procedures were performed under general anesthesia. Fat was harvested after infiltration of tumescent solution from donor sites selected based on availability, including the abdomen, posterior trunk, flanks or thighs. Fat was manually aspirated using a 10 mL syringe and liposuction cannula and processed by gauze rolling to remove excess fluid and oil [9].

**Figure 1.**
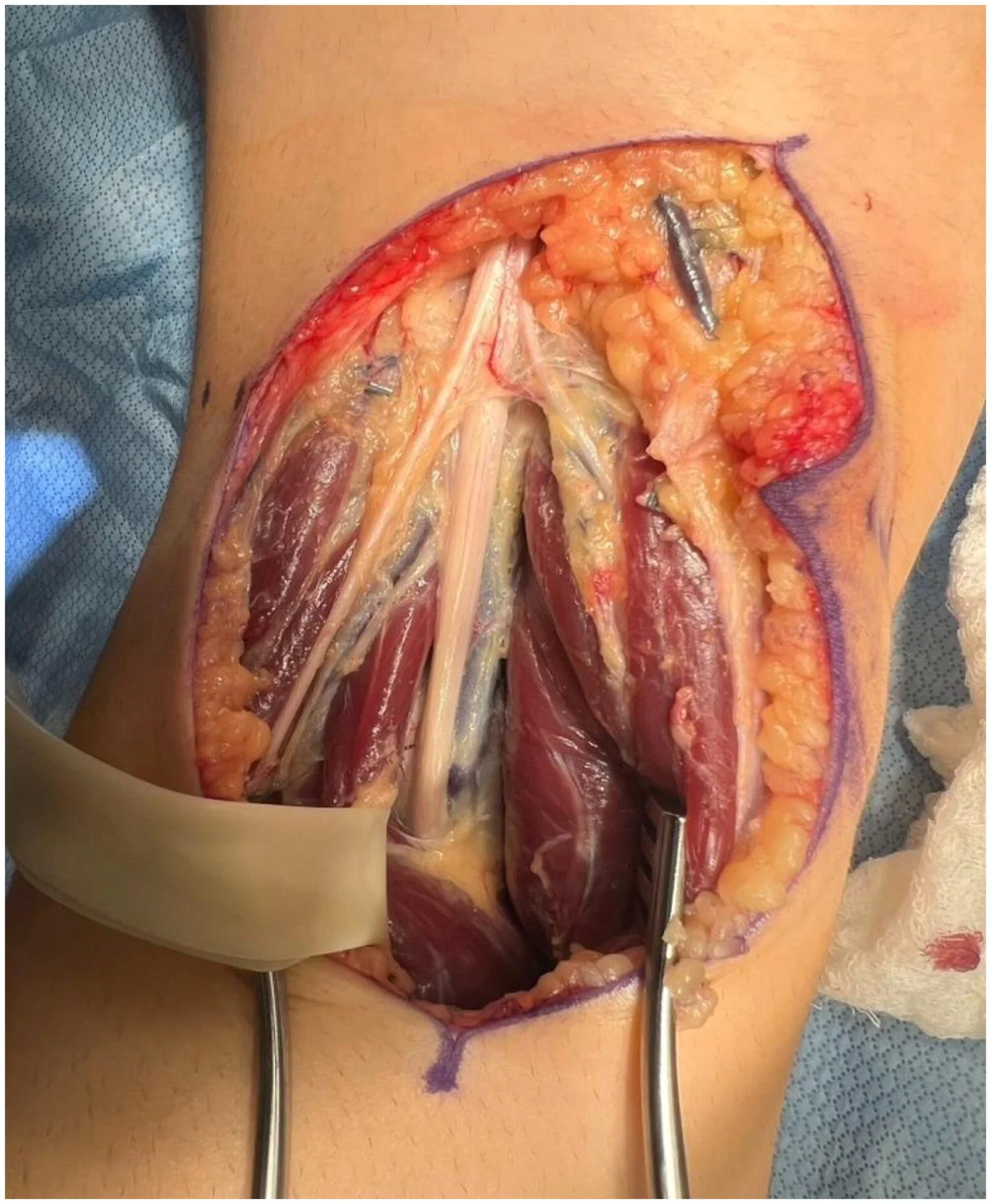
Intraoperative view of the tibial nerve (TN) at the level of the soleal sling. The TN is exposed within the deep posterior compartment after division of the compressive surrounding fascia.

**Figure 2.**
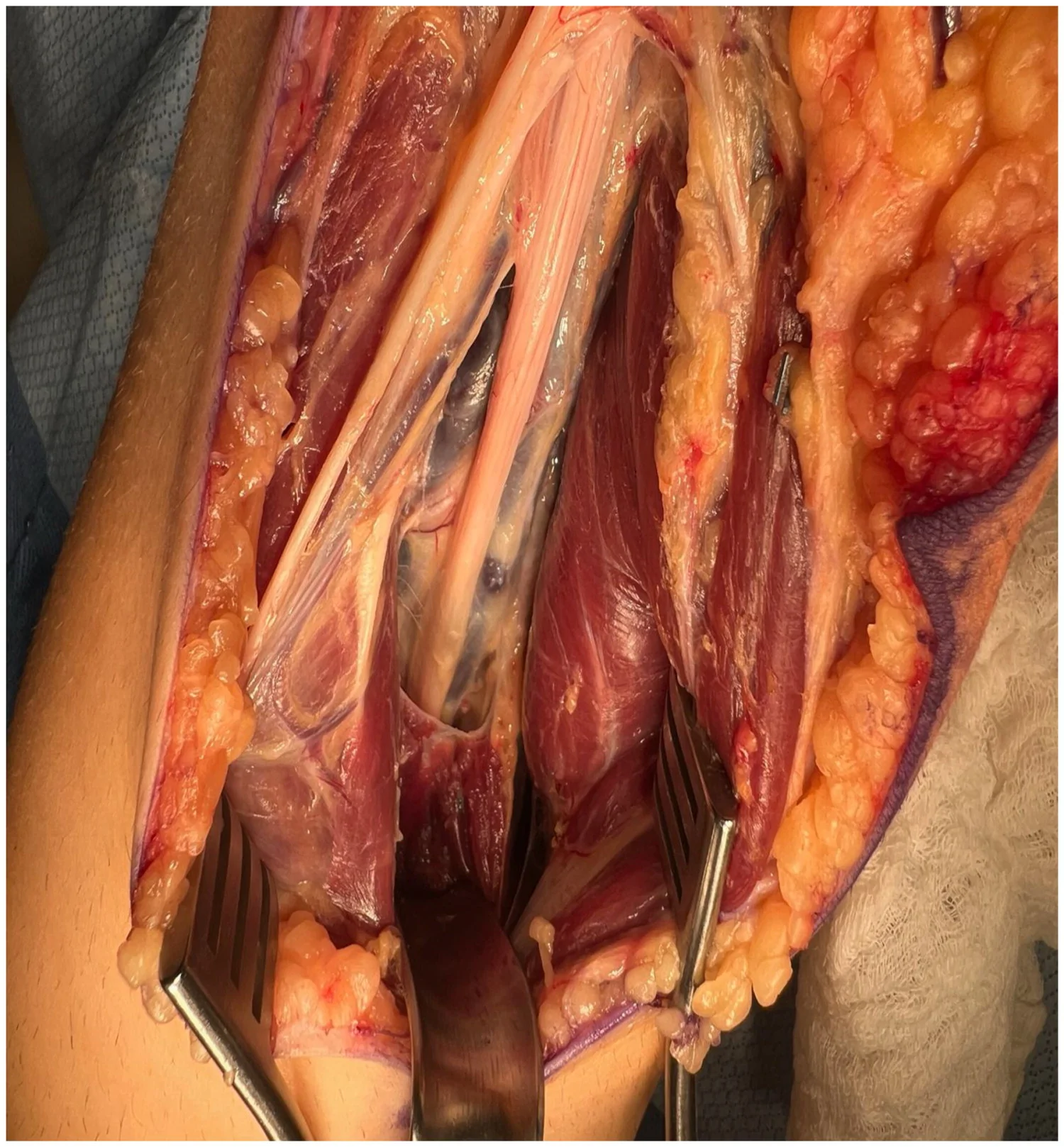
Close-up intraoperative view of the TN at the level of the soleal sling. The TN is visualized beneath the fibrous soleal sling within the deep posterior compartment prior to decompression.

**Figure 3.**
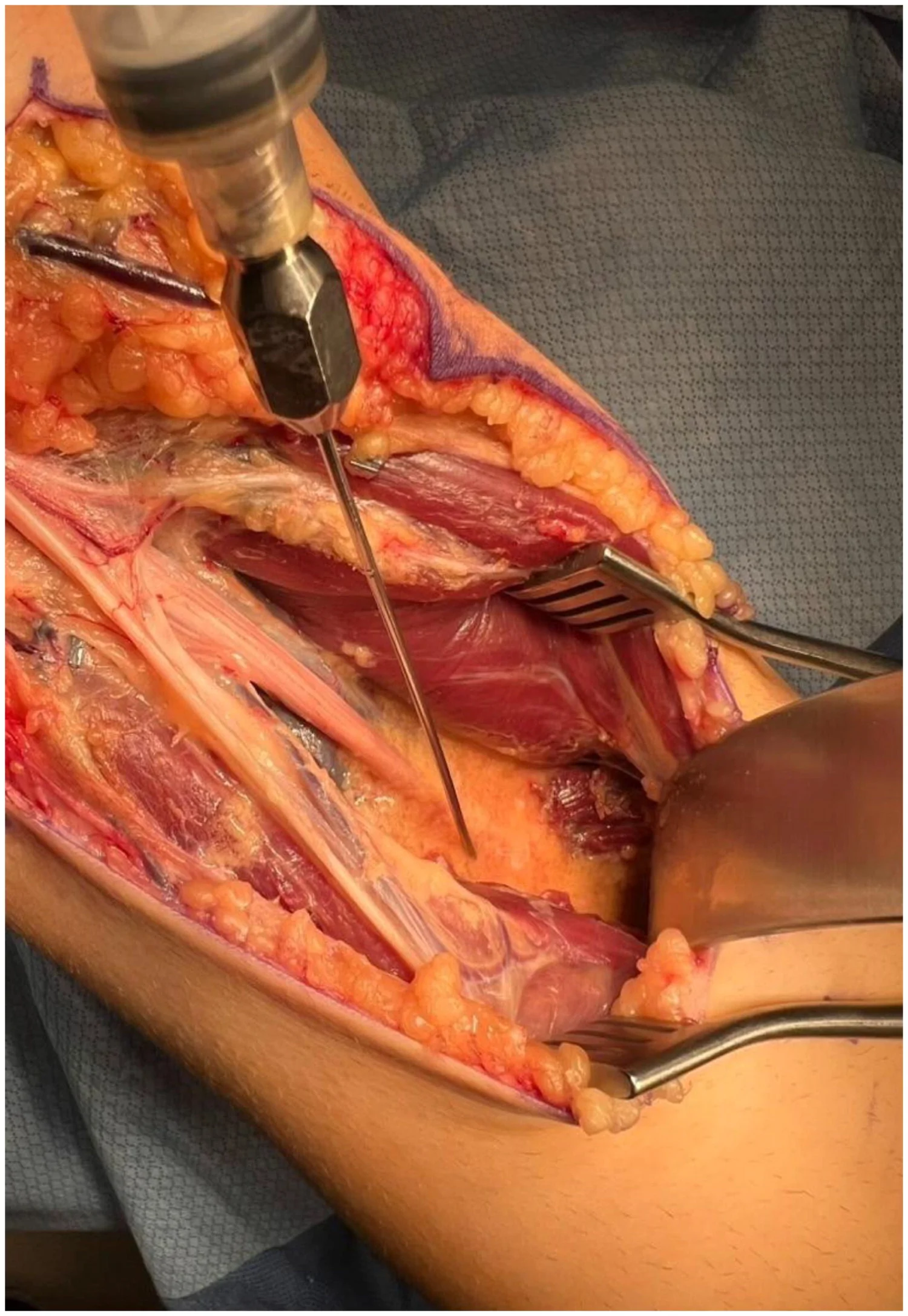
Intraoperative placement of autologous fat graft (AFG) following TN decompression. A cannula is introduced to deliver AFG around the decompressed TN at the level of the soleal sling to provide protective cushioning and reduce postoperative adhesions.

**Figure 4.**
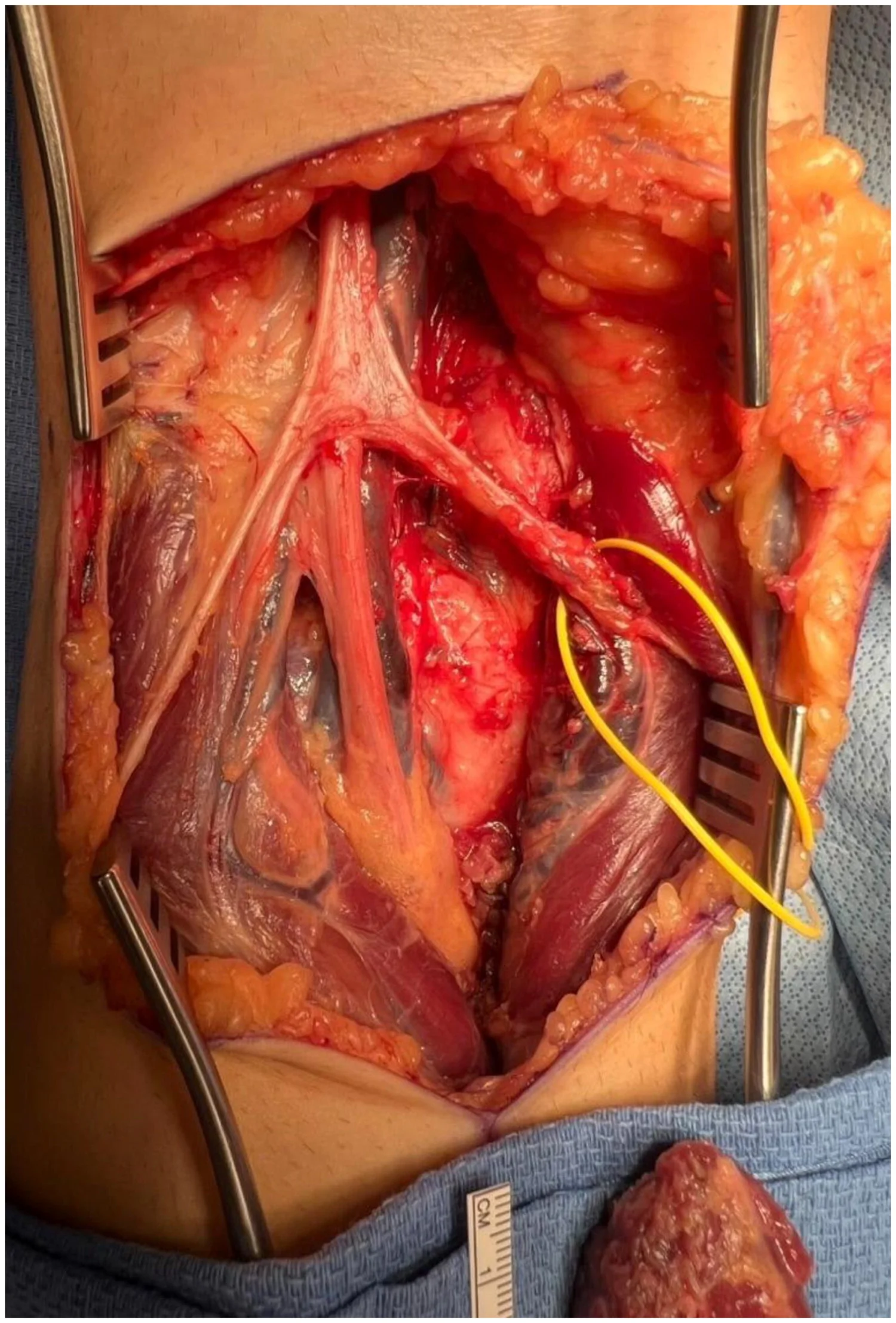
Final intraoperative view following TN decompression and AFG. After soleal sling release and neurolysis, AFG was placed circumferentially around the TN and along the decompression site and secured with fibrin glue.

Through a posterior proximal calf approach in the prone position, the gastrocnemius raphe was divided to expose the TN. Neurolysis was performed by releasing the fascia deep to the soleus muscle at the level of the soleal sling. Following decompression, AFG was injected circumferentially around the exposed TN along the entire length of release. Fibrin glue was applied to maintain graft position. Representative intraoperative images from patient 2 demonstrate AFG placement around the TN following decompression (Figures 1–4). The wound was closed in layers over a drain.

### Follow-up and outcome assessment

Patients were restricted to touchdown weight-bearing for three weeks postoperatively with early ankle and knee range of motion encouraged. Follow-up duration and number of visits were recorded. Clinical follow-up was defined as the interval from surgery to the last documented postoperative clinical visit. Imaging follow-up, when available, was reported separately. Clinical outcomes included symptom evolution, return to activity, complications and changes in PROMIS scores when available.

Continuous variables are presented as median (range), and categorical variables as frequency (percentage). No inferential statistical analyses were performed because of the descriptive nature and small sample size of this case series.

## Results

### Cohort characteristics

Five patients (seven limbs) underwent soleal sling release with adjunctive AFG. Baseline demographic, clinical characteristics and limb-level clinical and operative characteristics are summarized in Tables 1–3. All patients had concomitant PAES. Median age at surgery was 19.0 years (range 17–28), and median body mass index was 22.9 kg/m^2^. All patients were White, non-Hispanic and non-smokers.

**Table 1.** Limb-level clinical and operative characteristics.

Patient	Operated limb	Age	Sex	BMI	Relevant history	Laterality/rationale	Key workup	Volume AFG (mL)	Donor site
1	Left	27	F	21.9	Prior contralateral decompression; hydrodissection	Bilateral; staged left procedure	Dynamic US; compartment pressures not elevated	30	Abdomen
2	Left	16	M	22.9	Prior left fasciotomy; failed Botox	Bilateral; left treated first	Dynamic US; MRI	10	Flank
3	Left	19	F	22.3	Failed physical therapy	Bilateral, L > R; left treated first	Normal MRI/pressures; dynamic US; Tinel	10	Abdomen
3	Right	19	F	22.3	Failed physical therapy	Staged contralateral procedure	Clinical PAES/SSS; dynamic US	10	Abdomen
4	Left	22	F	32.3	Prior bilateral fasciotomies; Botox	Bilateral, L > R; left treated first	Dynamic US; CPN Tinel	10	Flank
4	Right	22	F	32.3	Prior bilateral fasciotomies; Botox	Staged contralateral procedure	Dynamic US	10	Thigh
5	Left	17	F	26	Failed PT/gait retraining; CPN compression	Predominantly left-sided symptoms	Dynamic US; CPN Tinel	10	Posterior trunk

AFG: autologous fat grafting; BMI: body mass index; Botox: botulinum toxin injection; CPN: common peroneal nerve; L: left; PT: physical therapy.

**Table 2.** Patient demographics.

Characteristic	Frequency
Age at the time of surgery (years)^a^	19.0 (17.0–28.0)
Gender	
Female	4 (80.0)
Male	1 (20.0)
Race	
White	5 (100.0)
BMI (kg/m^2^)	22.9 (22–32.3)

^a^ Reported as median (min–max).

**Table 3.** Clinical characteristics (statistics are reported per operated limb (*n* = 7)).

Characteristic	Frequency
Previous treatments	
Botox	4 (57.1)
Nerve hydrodissection	1 (14.3)
Operative compartment release	5 (71.4)
Physical therapy	3 (42.9)
Symptom duration (years)^a^	11.0 (0.3–9.0)
Length of surgery (minutes)^a^	115 (66–144)

^a^ Reported as median (min–max).

Median duration of symptoms prior to surgery was 1 year (range 0.3–9). Prior treatments included botulinum toxin injections (*n* = 4), nerve hydrodissection (*n* = 1) and previous compartment releases (*n* = 5). These patient characteristics are summarized in Table 3. Limb-specific pain scores were not consistently documented in the retrospective record.

### Operative characteristics

Median operative time was 115 min (range 66–144). All patients had a length of hospital stay of one day. Fat graft volumes ranged from 10 to 30 mL. Donor sites included abdomen (*n* = 3), posterior trunk (*n* = 1), flank (*n* = 2) and thigh (*n* = 1). All operated limbs underwent TN neurolysis at the soleal sling, popliteal artery exploration/decompression, posterior compartment release, plantaris resection and adjunctive AFG. Additional muscle debulking or partial denervation was performed based on intraoperative findings.

No intraoperative complications were observed.

### Clinical outcomes

Following surgery, most patients (*n* = 4) reported early improvement in exertional calf pain and sensory symptoms within 1–3 months postoperatively, although outcomes were heterogeneous. Three patients resumed full athletic activity within three months.

One patient reported recurrent bilateral exertional calf pain at 10 months postoperatively, with elevated compartment pressures suggesting a possible compartment-related component. Ultrasound-guided nerve hydrodissection provided partial symptom relief. No graft-related complications, wound complications or infections were observed.

PROMIS outcomes are summarized in Table 4. PROMIS Physical Function and Pain Interference outcomes were collected at variable postoperative timepoints and were patient-level rather than limb-specific measures. Paired PROMIS Pain Interference scores were available for all five patients, while paired PROMIS Physical Function scores were available for four patients. Pain Interference improved in three patients and worsened in two patients, with changes ranging from −6.5 to +6.7. Physical Function improved in three of four patients with paired data and decreased slightly in one patient, with changes ranging from −1.0 to +17.0. Because PROMIS data were not collected at standardized intervals and were not limb-specific, these results were interpreted descriptively.

**Table 4.** PROMIS patient-reported outcomes.

Patient	Baseline pain interference	Follow-up pain interference	Change	Baseline physical function	Follow-up physical function	Change
1	65.2	67.2	+2.0 worse	36.5	53.5	+17.0 improved
2	60.3	67.0	+6.7 worse	37.5	36.5	−1.0 worse
3	57.9	52.8	−5.1 improved	46.8	n/a	n/a
4	60.3	53.85	−6.45 improved	41.2	42.0	+0.8 improved
5	60.4	54.64	−5.76 improved	41.2	51.2	+10.0 improved

PROMIS scores were collected at the patient level and were not limb-specific. Follow-up timepoints varied across patients. For patients who underwent staged bilateral procedures, follow-up PROMIS scores reflect patient-level status after the listed procedures.

Median clinical follow-up duration was 7 months (range 3–14 months). Clinical follow-up was calculated using the last documented postoperative clinical visit; imaging follow-up was reported separately when available.

#### Patient 1

A 28-year-old female with a BMI of 22.0 presented with a nine-year history of bilateral PAES and TN compression. She reported persistent medial calf pain rated 6/10 on the visual analog scale (VAS) at baseline and constant plantar numbness that significantly limited physical activity to less than five minutes of exertion. Symptoms were exacerbated by walking, running and prolonged standing. Relevant history included prior fasciotomies and prior contralateral decompression, after which she developed scar-related traction neuritis that improved with hydrodissection. Dynamic point-of-care ultrasound demonstrated popliteal artery occlusion with resisted plantarflexion bilaterally, complete on the right and partial on the left. Compartment pressures were not elevated. The right side was initially more symptomatic and had been treated previously; she subsequently underwent left-sided surgery for persistent contralateral symptoms.

She underwent left-sided popliteal artery decompression, plantaris tendon resection, partial myomectomy of the medial gastrocnemius muscle, and TN neurolysis at the level of the soleal sling. Thirty milliliters of abdominal AFG were applied circumferentially around the exposed TN following decompression.

At 3 weeks postoperatively, she reported substantial reduction in calf pain and improved plantar sensation. Progressive weight-bearing and range-of-motion exercises were initiated. At 3 months, exertional pain had resolved completely, and she resumed unrestricted physical activity without limitation. At 6 months, she remained symptom-free with no recurrence of medial calf pain or sensory disturbance.

At 2-year imaging follow-up, bilateral MR neurography performed at that time demonstrated preserved perineural fat planes around the decompressed TN without neuroma formation, focal signal abnormality or recurrent compression. MR neurography of the contralateral limb demonstrated more extensive perineural soft tissue signal changes compared to the operated limb; this observation was descriptive and not formally quantified.

#### Patient 2

A 17-year-old male with a BMI of 22.9 presented with a six-year history of bilateral PAES and exertional lower extremity symptoms. He described deep posterior calf pain beginning within seconds of exertion, accompanied by cramping and intermittent paresthesias in both feet. Symptoms limited participation in sports and daily activities. Prior left-sided fasciotomy and botulinum toxin injections had failed to provide durable relief.

Dynamic ultrasound demonstrated bilateral popliteal artery occlusion with plantarflexion, and prior MRI reportedly showed bilateral popliteal occlusion without a fixed anatomic abnormality. Given bilateral disease, a staged approach was planned, and the left side was selected first because it had already undergone fasciotomy without symptomatic relief.

He underwent left-sided popliteal artery decompression, partial excision of the medial gastrocnemius muscle, plantaris resection, TN neurolysis and placement of 10 mL of bilateral flank-derived AFG.

At 1 month postoperatively, he reported complete resolution of calf pain and paresthesias. By 2 months, he had returned to full athletic participation, including competitive soccer. At 4 months, he noted mild recurrence of calf discomfort with high-intensity activity.

At 10 months, he reported persistent or recurrent bilateral exertional calf pain, including pain at rest, which limited sports participation. Repeat compartment pressure testing demonstrated elevated pressures, and symptoms were felt to be at least partly compartment-related. Ultrasound-guided hydrodissection provided partial symptom relief.

No surgical complications, wound issues or graft-related adverse events were observed.

#### Patient 3

A 19-year-old female with a BMI of 22.5 presented with a 3.5-month history of bilateral posterior calf pain and plantar foot and ankle paresthesia’s, worse on the left side. She described sharp and aching pain during athletic activities, particularly soccer and basketball, with associated calf firmness after exertion. She had tried physical therapy once without symptom resolution. Compartment pressure testing and MRI were unremarkable. Dynamic ultrasound demonstrated popliteal artery compression with resisted plantarflexion. Clinical examination revealed focal tenderness and a positive Tinel’s sign at the soleal sling bilaterally. Given bilateral symptoms with greater left-sided severity, the left side was treated first.

She underwent left-sided decompression with application of 10 mL of abdominal AFG. Four months later, due to persistent contralateral symptoms, she underwent staged right-sided decompression with identical adjunctive fat grafting.

At 2 months following the first procedure, resting pain and plantar paresthesias had resolved, and exertional symptoms were markedly improved. After the right-sided surgery, she experienced mild postoperative swelling and intermittent plantar tingling, though symptoms were less severe than preoperatively. Over the subsequent five months, she reported progressive improvement, return to athletic participation, and resolution of sharp exertional pain. She did not require additional interventions.

#### Patient 4

A 23-year-old female with a BMI of 32.3 presented with bilateral exertional calf pain, diffuse foot numbness and episodic foot drop triggered by ambulation or light activity. Symptoms were worse on the left side, but limb-specific pain scores were not documented. Pain episodes lasted approximately 45 min and could occur with ambulation alone. Symptoms persisted despite prior superficial and deep posterior compartment fasciotomies. Targeted botulinum toxin injections applied bilaterally resulted in temporary improvement lasting approximately three to four months. Examination demonstrated well-healed fasciotomy incisions, soft compartments, preserved ankle dorsiflexion and plantarflexion strength, intact light-touch sensation, and bilateral Tinel’s signs over the common peroneal nerve at the fibular neck. Functional PAES was confirmed by history and dynamic ultrasound with resisted plantarflexion.

She underwent staged bilateral decompressions beginning with the more symptomatic left side. Ten milliliters of flank-derived AFG were placed during the first procedure, followed by staged right-sided decompression with 10 mL of thigh-derived AFG.

At 2 months following the first surgery, exertional calf pain, numbness and foot drop had resolved, permitting return to athletic activity including field hockey. Following the second procedure, she experienced mild posterior knee discomfort and transient quadriceps weakness, which improved with physical therapy. Overall, she reported marked improvement compared to baseline, with no recurrence of foot drop episodes during follow-up.

#### Patient 5

A 19-year-old female with a BMI of 25.5 presented with a one-year history of predominantly left-sided lower extremity symptoms, including lateral leg pain, left foot paresthesias, ankle pain, weakness with running and intermittent foot drop. Symptoms were constant but worsened with activity and she reported firmness of the left lower extremity after exertion. Physical therapy and gait retraining did not resolve her symptoms. She had no relevant past medical history, no active medications and no documented allergies. Examination demonstrated a positive Tinel’s sign over the left common peroneal nerve. Dynamic ultrasound of the bilateral popliteal fossae demonstrated popliteal artery occlusion with resisted plantarflexion bilaterally. Initial assessment included left common peroneal nerve compression and bilateral PAES.

She initially pursued conservative management for PAES. Given persistent exertional symptoms, she later underwent operative treatment including popliteal artery decompression, posterior fasciotomies, TN neurolysis, revision common peroneal nerve decompression and placement of 10 mL posterior trunk-derived AFG around the TN. The documented rationale for AFG was to reduce adhesions and traction neuritis around the TN.

At 1 month postoperatively, she reported reduction in posterior knee and calf pain with decreased frequency of numbness episodes. At 3 months, functional gains were maintained, though intermittent activity-related tightness persisted. No wound complications, infections or graft-related adverse events were observed during follow-up.

## Discussion

This case series describes five patients (seven limbs) with SSS who underwent TN decompression with adjunctive AFG. The principal findings are that AFG was technically feasible, well tolerated, and not associated with graft-related complications.

Early postoperative improvement in exertional calf pain and sensory symptoms was observed in most patients; however, all patients also underwent concomitant PAES correction and additional simultaneous procedures, including posterior compartment release, plantaris resection and varying degrees of muscle debulking or partial denervation. One patient also underwent revision common peroneal nerve decompression. Therefore, postoperative improvement reflects a broader decompressive surgical strategy, and the independent contribution of AFG cannot be determined. This study should be interpreted primarily as an early technical and short-term safety experience rather than evidence of AFG efficacy.

Proximal TN compression at the soleal sling is increasingly recognized as a cause of exertional lower leg pain and plantar neuropathic symptoms. Its anatomical course beneath the fibrous arch of the soleus muscle creates a potential entrapment site, particularly in physically active individuals or those with anatomical variation. Previous studies have documented this entrapment site through cadaveric [1], imaging [10] and surgical observations [2], supporting its clinical relevance. However, as highlighted in a recent narrative review, SSS lacks standardized diagnostic criteria, and the existing literature remains limited by small case series, heterogeneous diagnostic workups, subjective outcome measures and limited follow-up [11]. Therefore, the findings of the present series should be interpreted within the broader context of an evolving and incompletely standardized clinical entity.

In our case series, patients commonly experienced prolonged symptom duration prior to surgical intervention. The median time from symptom onset to surgery was 1 year, reflecting a diagnostic delay that has been described in peripheral nerve entrapment syndromes [2].

Standard imaging modalities and compartment testing were frequently inconclusive, emphasizing the importance of clinical suspicion and dynamic assessment in the evaluation of exertional neurovascular complaints.

All patients underwent decompression of the TN at the soleal sling with resection of contributing musculotendinous structures and circumferential application of AFG. A posterior surgical approach was favored over a medial approach to facilitate comprehensive exposure of the popliteal fossa and distal TN [12]. This positioning allowed extensive fascial release and three-dimensional assessment of potential compression sites while maintaining a relatively limited incision.

Postoperative perineural fibrosis and adhesion formation remain recognized challenges in peripheral nerve surgery [13]. The intended rationale for AFG in this series was to provide a mechanical interface around the decompressed TN, improve nerve gliding, reduce friction against surrounding tissues and potentially limit postoperative adhesion formation or traction neuritis. Fat grafts may function as a spacer, reducing friction and external compression, as described by Vaienti et al. [5]. Additionally, adipose tissue contains mesenchymal stem cells with angiogenic and immunomodulatory properties that may support nerve healing and microvascularization [6]. Krześniak and Noszczyk demonstrated favorable outcomes using fat grafting in recurrent compressive neuropathies, including revision carpal tunnel surgery [7]. Experimental data further support the role of adipose-derived stem cells in modulating inflammation and reducing perineural adhesion formation [6,14,15]. Nevertheless, no established indication currently exists for AFG in SSS, and this series does not determine whether AFG should be used routinely during soleal sling release.

All patients in this series had concomitant PAES, highlighting the potential overlap between vascular and neural compression within the popliteal fossa [16]. This coexistence is clinically important because PAES correction alone may improve exertional calf pain, making attribution of postoperative symptom improvement to TN decompression or AFG difficult. In the present series, AFG was used as a routine adjunct during soleal sling decompression by the senior author, with the intended rationale of improving nerve gliding, reducing adhesion formation and limiting traction neuritis. The absence of graft-related complications supports its technical feasibility and short-term safety, but comparative studies are required to determine whether it provides incremental benefit beyond decompression alone.

This study has limitations. The small sample size, retrospective design and absence of a control group limit generalizability and preclude causal inference. Although PROMIS outcomes were available for most patients, scores were collected at variable postoperative timepoints and were patient-level rather than limb-specific measures, limiting their interpretive value. In addition, all patients underwent concomitant PAES correction and additional simultaneous decompressive procedures, which represents a major confounder when interpreting postoperative symptom improvement. The proposed rationale for AFG includes reducing perineural fibrosis, recurrent tethering and traction neuritis; however, these are long-term processes that cannot be evaluated with a median clinical follow-up of 7 months. One patient reported persistent or recurrent bilateral exertional calf pain at 10 months, with elevated compartment pressures suggesting a possible compartment-related component. Therefore, longer follow-up is needed to assess recurrence after combined decompression procedures.

## Conclusions

Adjunctive AFG during TN decompression for SSS was technically feasible and not associated with graft-related complications in this small case series. Because all patients underwent concomitant PAES correction, postoperative symptom improvement cannot be attributed to AFG alone. While the independent effect of fat grafting cannot be determined from this study, its consistent use and absence of graft-related complications support further investigation. Prospective comparative studies are warranted to evaluate whether AFG provides additional benefit beyond decompression alone and to clarify its potential role in reducing perineural scarring.

## Data Availability

The data supporting the findings of this study are available from the corresponding author upon reasonable request. Data are not publicly available due to patient privacy considerations.
